# Supplementary figures and images for: Demographic and Socioeconomic Disparity in Knowledge About Tuberculosis in Inner Mongolia, China
Source: J Epidemiol. 2015 Apr 5;25(4):312–20. doi: 10.2188/jea.JE20140033 (PMC4375286; doi:10.2188/jea.JE20140033)

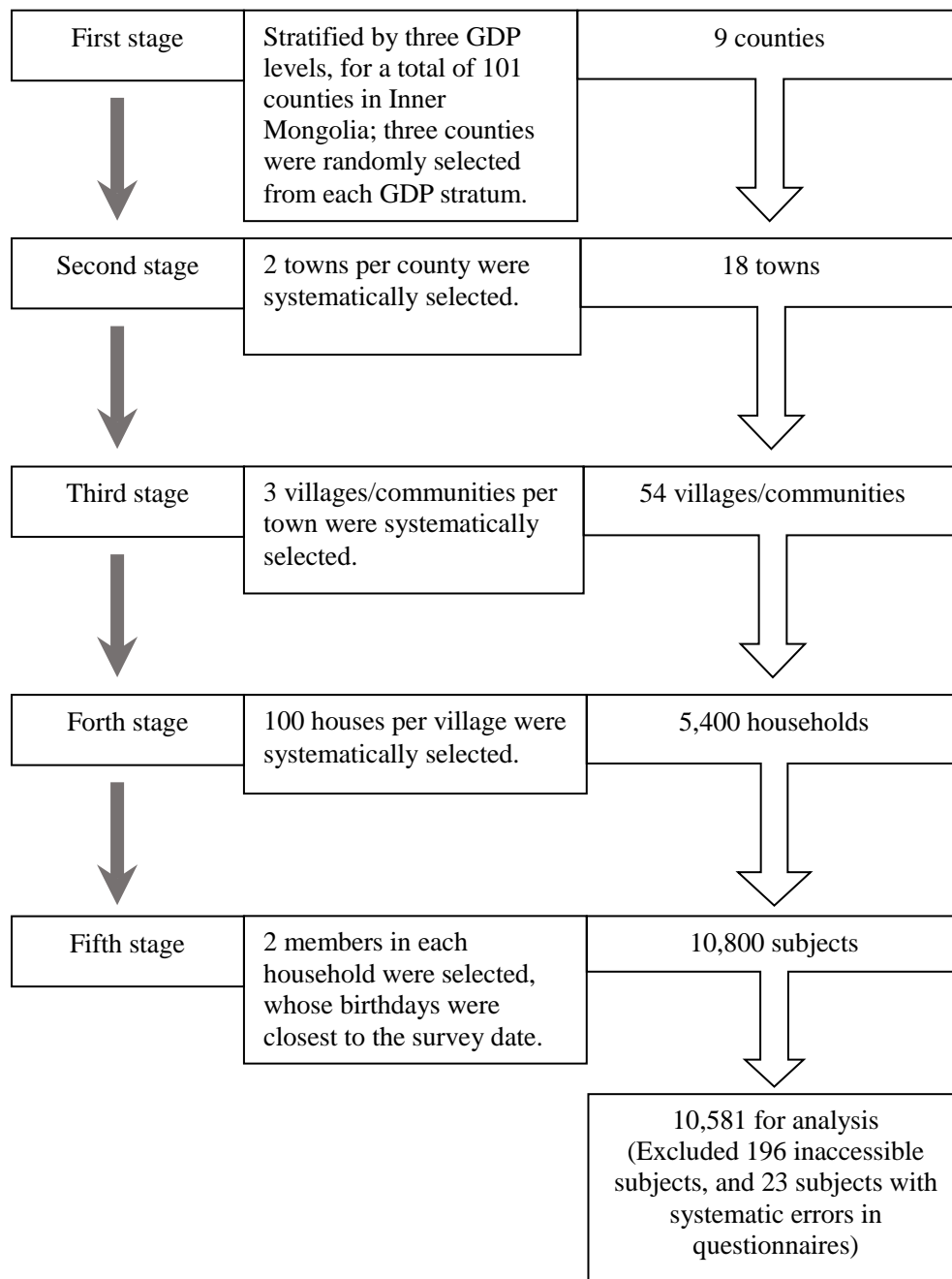

**eFigure1. Flowchart on sampling process**

Supplement: eFigure 1. [file je-25-312-s001.pdf]
