# Supplementary material for: Demographic and Socioeconomic Disparity in Knowledge About Tuberculosis in Inner Mongolia, China
Source: J Epidemiol. 2015 Apr 5;25(4):312–20. doi: 10.2188/jea.JE20140033 (PMC4375286; doi:10.2188/jea.JE20140033)
Supplement: eTable 1. [file je-25-312-s002.pdf]

eTable 1. Sample design weights and non-response weight

Sampling frame (2000 Inner Mongolia Census)						Sampling units in each stage					Sampling fractions in each stage, weights, and weighted sample							
County code	Town (Ti)	Village or Community (Vi)	Household (Hi)	Population (Ni)	Proportion to GDP group population (Pi)	Towns/ county (ti)	Villages/ town (vi)	Household/ village (hi)	Subject/ household (si)	Total subjects (ni)	ft ti/Ti	fv vi/(Vi/Ti)	fh hi/(Hi/Vi)	fs si/(Ni/Hi)	Design weight DW= 1/(ft*fv*fh*fs)/Pi	Eligible subjects nj	Weight with non-response DW'= DW*ni/nj	Weighted sample ni*DW'
9100	9	96	88,679	266,888	0.110	2	3	100	2	1200	0.22	0.28	0.11	0.66	2,023.39	1,132	2,144.93	2,428,063
9140	13	133	125,661	382,659	0.110	2	3	100	2	1200	0.15	0.29	0.11	0.66	2,901.09	1,197	2,908.36	3,481,312
9215	8	82	20,560	67,882	0.078	2	3	100	2	1200	0.25	0.29	0.40	0.61	729.07	1,192	733.96	874,886
9211	11	63	44,460	146,808	0.071	2	3	100	2	1200	0.18	0.52	0.14	0.61	1,729.38	1,191	1,742.44	2,075,252
1374	16	282	88,050	341,574	0.071	2	3	100	2	1200	0.13	0.17	0.32	0.52	4,023.69	1,166	4,141.02	4,828,428
9134	9	299	87,784	71,186	0.078	2	3	100	2	1200	0.22	0.09	0.34	2.47	764.56	1,191	770.33	917,469
9123	7	119	52,773	276,156	0.071	2	3	100	2	1200	0.29	0.18	0.23	0.38	3,253.08	1,143	3,415.30	3,903,691
9272	8	128	21,277	172,867	0.078	2	3	100	2	1200	0.25	0.19	0.60	0.25	1,856.64	1,186	1,878.56	2,227,966
9154	9	134	76,153	284,279	0.110	2	3	100	2	1200	0.22	0.20	0.18	0.54	2,155.23	1,183	2,186.21	2,586,281
Total	90	1,336	605,397	2,010,299		18	27	900	18	10,800						10,581		23,323,349
